# Improved method for predicting linear B-cell epitopes

**DOI:** 10.1186/1745-7580-2-2

**Published:** 2006-04-24

**Authors:** Jens Erik Pontoppidan Larsen, Ole Lund, Morten Nielsen

**Affiliations:** 1 Center for Biological Sequence Analysis, BioCentrum-DTU, Building 208, Technical University of Denmark, DK-2800 Kgs. Lyngby, Denmark

## Abstract

**Background:**

B-cell epitopes are the sites of molecules that are recognized by antibodies of the immune system. Knowledge of B-cell epitopes may be used in the design of vaccines and diagnostics tests. It is therefore of interest to develop improved methods for predicting B-cell epitopes. In this paper, we describe an improved method for predicting linear B-cell epitopes.

**Results:**

In order to do this, three data sets of linear B-cell epitope annotated proteins were constructed. A data set was collected from the literature, another data set was extracted from the AntiJen database and a data sets of epitopes in the proteins of HIV was collected from the Los Alamos HIV database. An unbiased validation of the methods was made by testing on data sets on which they were neither trained nor optimized on. We have measured the performance in a non-parametric way by constructing ROC-curves.

**Conclusion:**

The best single method for predicting linear B-cell epitopes is the hidden Markov model. Combining the hidden Markov model with one of the best propensity scale methods, we obtained the BepiPred method. When tested on the validation data set this method performs significantly better than any of the other methods tested. The server and data sets are publicly available at .

## Background

Vaccines have mostly been composed of killed or attenuated whole pathogens. For safety reasons, however, it could be desirable to use peptide vaccines that are able to generate an immune response against a given pathogen [1]. Such vaccines could contain peptides representing linear B-cell epitopes from the proteins of the pathogen. Hughes et al. [2] used linear B-cell epitopes to induce protective immunity in mice against *P. aeruginosa*. By immunizing animals, synthetic peptides containing linear B-cell epitopes can also be used to raise antibodies against a specific protein, which e.g. can be used in screening assays or as diagnostic tools [3].

B-cell epitopes are parts of proteins or other molecules that antibodies (made by B-cells) bind. Most protein epitopes are composed of different parts of the polypeptide chain that are brought into spatial proximity by the folding of the protein. These epitopes are called discontinuous, but for approximately 10% of the epitopes, the corresponding antibodies are cross-reactive with a linear peptide fragment of the epitope [4]. These epitopes are denoted linear or continuous and are mainly composed of a single stretch of the polypeptide chain.

Even though linear B-cell epitopes thus are of limited relevance in the detailed understanding of a humoral immune response, identification of such linear peptide segments will often be the initial step in the search for antigenic determinants in pathogenic organisms. The traditional experimental peptide scanning approach is clearly not feasible on a genomic scale. Prediction methods are very cost effective and reliable methods for predicting linear B-cell epitopes would therefore be a first step in guiding a genome wide search for B-cell antigens in pathogenic organism.

The classical way of predicting linear B-cell epitopes is by the use of propensity scale methods. These methods assign a propensity value to every amino acid, based on studies of their physico-chemical properties. Fluctuations in the sequence of prediction values are reduced by applying a running average window. This prediction procedure was first developed by Hopp and Woods [5].

Pellequer et al. [4] compared several propensity scale methods using a data set of 14 epitope annotated proteins. They found that applying the scales by Parker et al. [6] (hydrophilicity), Chou and Fasman [7] and Levitt [8] (secondary structure) and by Emini et al. [9] (accessibility) gave slightly better results than the other scales tested.

Alix [10] developed a program called PEOPLE, which predicts the location of linear B-cell epitopes using combinations of propensity scale methods. Odorico [11] have developed a program, BEPITOPE, for predicting the location of linear B-cell epitopes using propensity scale methods.

Recently, Blythe and Flower [12] studied the performance of many propensity scale methods and found that even the best methods predict only marginally better than a random model. They made a thorough study using a data set of 50 epitope mapped proteins from the AntiJen web page [13].

In this study, we have developed a novel method for predicting linear B-cell epitopes, BepiPred, which is found to perform both significantly better than random predictions as well as significantly better than a number of tested propensity scales.

Even though the present method is a significant improvement over earlier methods for predicting linear B-cell epitopes, it still has major limitations. There is a need for further improvements in predictive power before such systems become generally useful to provide reliable predictions of B-cell epitopes.

## Results

### Predictions by propensity scale methods

We first tested a number of propensity scale methods on the Pellequer data set [14]. For every scale and window size, a ROC-curve and area under it, the *A*_*roc*_-value, was calculated as a measure of the prediction accuracy. 1000 bootstrap samples were drawn from the predictions in order to estimate the standard error of the *A*_*roc*_-value, . The best scale was found to be the one by Levitt [8] (window size of 11, *A*_*roc *_= 0.658 ± 0.013). This method with will be denoted Levitt. The second best scale is the scale by Parker et al. [6] (window size 9, *A*_*roc *_= 0.654 ± 0.013), denoted Parker. The other scales, that were tested, did not perform as well as the scales by Parker et al. [6] and Levitt [8].

Performing a permutation experiment 1000 times, we estimated the P-value for the hypothesis that a method performs like a random model, where the alternative hypothesis is that it performs better than a random model. The resulting P-values for Parker and Levitt were both below 0.1%.

### Predictions by hidden Markov models

Experiments were conducted in which hidden Markov models (HMMs) were used for the prediction of the location of linear B-cell epitopes. The methods were build from positive windows extracted from the AntiJen data set. The HMMs were tested on the Pellequer data set to find the optimal parameters. Different sizes of the extracted peptide windows, different weights of pseudo count correction for estimating the amino acid frequencies and different sizes of the smoothing window were tested. For the best method, the size of the extracted windows was found to be 5, the size of the smoothing window was 9 and the pseudo-count correction was 10^7^. The performance of the method on the Pellequer data set was *A*_*roc *_= 0.663 ± 0.012. This method with these parameters will be denoted HMM.

### Combining methods

In order to make more accurate predictions, the hidden Markov model (HMM) was combined with one of the two best propensity scale methods (Parker and Levitt). The combinations were done as weighted sums of normalized prediction values. The sum of the weights on the two methods was kept equal to one and different weight-pairs were tested. The Pellequer data set was used to optimize the parameter values. The combination methods with the highest *A*_*roc*_-values were chosen for further comparisons and are shown in Table 1. The combinational method with the highest *A*_*roc*_-value is denoted BepiPred and it is the candidate method for predicting linear B-cell epitopes in this paper. It is a combination of HMM and Parker.

**Table 1:** Combinations of methods. Predictions on the Pellequer data set.

Name	Method 1	Method 2	Weight on method 1	*A*_*roc*_
BepiPred	HMM	Parker	0.60	0.671 ± 0.013
Comb2	HMM	Levitt	0.55	0.669 ± 0.013

### Validating the methods

**Figure 1:**
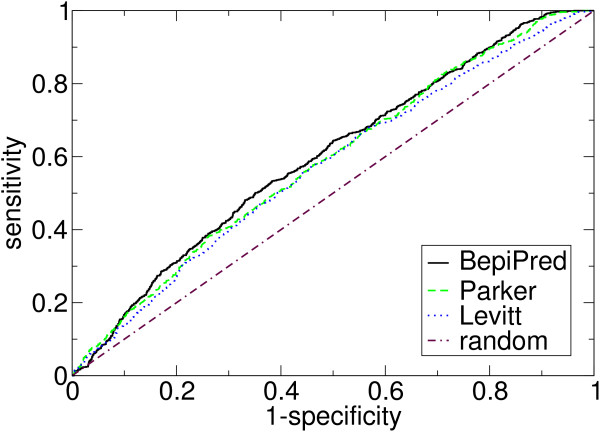
ROC-curves for selected methods validated on the HIV data set. See Table 3 for chosen points on the curves.

To make an unbiased validation of the methods, tests were performed on an independent data set, the HIV data set. The results are shown in Table 2. BepiPred is again seen to be the best method. ROC-curves for the selected methods are shown in Figure 1, and chosen values are given in Table 3.

**Table 2:** Validation of the methods on the HIV data set.

Method	*A*_*roc*_
BepiPred	0.600 ± 0.011
HMM	0.586 ± 0.011
Parker	0.586 ± 0.011
Comb2	0.584 ± 0.011
Levitt	0.572 ± 0.011

**Table 3:** Sensitivities for selected specificities (both in %) for some of the methods. The data is taken from their ROC-curves, shown in Figure 1. The methods were validated on the HIV data set.

specificity	sensitivity		
	BepiPred	Parker	Levitt
90	16.7	17.2	14.5
80	30.9	28.8	26.8
70	42.6	40.8	39.6
60	53.8	50.9	50.1

Paired t-tests were performed for the predictions on the HIV data set to determine if one method had a prediction accuracy that was significant higher than another. Table 4 shows that BepiPred was found to be significantly better than all other tested methods, and that HMM was not significantly better than Parker.

**Table 4:** P values (in %) for the comparisons of methods. If a P-value is below the chosen significance level of 5%, the alternative hypothesis, which is that the method to the left is more accurate than the method at the top, can be accepted. The methods were validated on the HIV data set.

	Levitt	Comb2	Parker	HMM
Comb2	0.33			
Parker	12.62	43.61		
HMM	2.60	20.15	45.86	
BepiPred	**0.04**	**0.14**	**1.90**	**0.13**

## Discussion

We have constructed a prediction method for linear B-cell epitopes using a hidden Markov model. Hidden Markov models have not been used for this specific purpose before.

Our method has a quite low sensitivity. One way of increasing the sensitivity is to lower the applied threshold, but that would also lead to a lower specificity. Pellequer et al.[14] showed that a reduction of over-predictions could be done by combining prediction curves, and further improvements of B-cell epitope prediction methods may be obtained using similar approaches.

Pellequer et al. [4] have made a comparison of several propensity scales using one of the data sets in the present study: the Pellequer dataset. They made a study applying some propensity scale methods to the data set and used a fixed threshold of 0.7 *s*, where *s *is the standard deviation of the prediction values. This threshold classified the predictions as positive or negative. They found that the predictions using the different scales were better than random, complying with the findings of the present study. They compared the scales on a data set consisting of nine of the sequences and found that the scales by Parker et al. [6], Chou and Fasman [7], Levitt [8] and of Emini et al. [9] gave slightly better results than the other scales tested.

In the present study, we found that for a similar data set, the scales that performed best were constructed by Levitt [8] and Parker et al. [6]. This corresponds well with the findings of Pellequer et al. [4].

Blythe and Flower [12] have found that even the best propensity scale methods perform only marginally better than a random model. They used a data set of 50 epitope mapped proteins from the AntiJen home page [13] and applied many propensity scale methods to the data.

Our permutation tests showed that the scales by Parker et al. [6] and Levitt [8] with their optimal window sizes were performing significantly better than random models.

We have tested several propensity scale methods and optimized their parameters in order to identify the best method. For the Pellequer data set, the best method was found to be the scale by [8] with a window size of 11. The second best propensity scale method was the scale by Parker et al. [6] with a window size of 7–11. This scale was intended to be used with a window size of 7 by the authors, which corresponds well with our findings.

## Conclusion

We present a novel method for predicting linear B-cell epitopes, BepiPred. It is a combination method, made by combining the predictions of a hidden Markov model and the propensity scale by Parker et al. [6]. We have tested different parameters in order to optimize the hidden Markov model and the propensity scale method.

We have tested the methods using the non-parametric ROC-curves and made an unbiased validation using a separate data set. We found that BepiPred had the highest prediction accuracy on the test data set, and it is shown to perform significantly better than all other methods tested on the validation data set. Comparing BepiPred with the best propensity scale methods on the validation data set, for a specificity of 80% the sensitivity for BepiPred, the scale by Parker et al. [6] and by Levitt [8] is 30.9%, 28.8% and 26.8%, respectively.

Future work could include using data from other sources, such as the Immune Epitope Database and Analysis Resource, IEDB [15], or the Epitome database of structurally inferred antigenic epitopes in proteins .

## Data sets

Three data sets of proteins with linear B-cell epitope annotation were used in these studies. All data sets were constructed by measuring the cross-reactivity between the intact protein and the peptide fragment [16].

### The Pellequer data set

A data set was used for the tests and optimization of the methods. Since this dataset was unavailable in an electronic form it was recreated by Lund et al. [17]. The epitope annotations were taken from Pellequer et al. [14] and references herein. An exception was the sequence of scorpion neurotoxin, in which the data was taken from [18]. This data set, denoted the Pellequer data set, contains 14 protein sequences and 83 epitopes. The epitope density is 0.34.

### The AntiJen data set

A second data set was used to train and build the hidden Markov model. This data set was extracted from the AntiJen database, formerly JenPep [13]. This data set, denoted the AntiJen data set, consists of 127 protein sequences, and the epitope density is 0.08. The proteins of this data set are not fully annotated, and the annotation for the non-epitope stretches is not known.

### The HIV data set

A separate data set was made allowing an unbiased validation of the methods. It consists of epitopes found in the proteins of HIV taken from the HIV Molecular Immunology Database of the Los Alamos National Laboratory [19]. The epitopes in this data set are overlapping to some degree. Therefore a procedure for determining more accurate borders of the minimal epitopes was applied to the epitopes. If a smaller epitope was contained as part of a larger epitope, the larger epitope was discarded from the data set. Two of the sequences had no assigned epitopes and were therefore discarded from the data set. The HIV data set consists of 10 protein sequences and the epitope density is 0.38.

## Methods

### Propensity scale methods

The propensity scale methods assign a propensity value to every amino acid of the query protein sequence. Fluctuations are reduced by applying a running mean window. In the N- and C- termini we used asymmetric windows to avoid discarding prediction examples. The scales used in this study are based on antigenicity [20], hydrophilicity [6], inverted hydrophobicity [21,22], accessibility [9] and secondary structure [7,8].

### Hidden Markov models

Let **i **= (*i*_1_, *i*_2_, ..., *i*_*w*_) denote a sequence of amino acids, which has been extracted from a protein sequence. Let *j *denote the position in this window, *j *= 1...*w*. On basis of **i**, the hidden Markov model predicts if the center position of the window is annotated as part of an epitope. In the N- and C-termini, parts of the extracted windows are exceeding the terminals. For these residues, the character 'X' is used, which does not count when the hidden Markov model is used for the predictions. The prediction score for a window is given by



which is the log odds of the residue at the center position of the window is being part of an epitope (Epitope model) as opposed to if it is occurring by chance (Random model).

To construct the Random model, background frequencies of the Swiss-Prot database [23], *q*_*i*_, is used. For the Epitope model, *p*_*i,j *_is the effective amino acid probability of having amino acid *i *at position *j *according to the model.

To calculate the values of *p*_*i,j*_, all windows, for which their center position is annotated as part of an epitope, are extracted from atraining data set. Again, if an extracted window exceeds the N or C terminal, the character 'X' is used, which does not count when calculating the parameters.

These extracted peptide windows form a matrix of aligned peptides of the width *w*. From this alignment, *p*_*i,j *_is calculated as the pseudo count corrected probability of occurrence of amino acid *i *in column *j*, estimated as in [24]. To make the pseudo count correction, pseudo count frequencies, *g*_*i,j*_, are calculated. They are given by



where *p*_*k,j *_is the observed frequency of amino acid *k *in column *j *of the alignment [25]. The variable *b*_*i,k *_is the Blosum 62 substitution matrix frequency, e.g. the frequency of which *i *is aligned to *k *[26].

To give an example of using (2), let the window size, *w *= 1. The model is then only covering residues, which are annotated as being part of linear B-cell epitopes. If the observed peptides consists of the following single amino acid sequences L and V, with the frequencies *p*_*L,1 *_= 0.5 and *p*_*V,1 *_= 0.5, then the pseudo-count frequency for e.g. I is given by



The effective amino acid frequencies are calculated as a weighted average of the observed frequency and the pseudo count frequency,



Here, *α *is the effective number of sequences in the alignment - 1, and *β *is the pseudo count correction [25], which is also called the weight on low counts. To finish the calculation example, let *β *be very large as it is in this work. Then *p*_*I,1 *_≈ *g*_*I,1 *_= 0.14.

Note that we shall use the term hidden Markov model throughout this work to refer to the weight matrix generated using (1). The parameters of the ungapped Markov model are calculated using a so-called Gibbs sampler, written by Nielsen et al. [24].

The result of applying (1) is a prediction score for every residue of the query sequence. To reduce fluctuations, a smoothing window is applied to every position. It is made asymmetric in the N- and C- termini in order to conserve prediction examples.

### ROC-curves

The result of applying a prediction method to a data set is a set of prediction examples, **x **= (*x*_1_, *x*_2_, ...,*x*_*N*_). Let *n *denote the residue number. Every *x*_*n *_consists of a target value and a predicted value. If the residue is annotated as part of an epitope, the target value is 1, zero otherwise. If asymmetric smoothing windows are used in the N- and C- termini, the variable *N *is equal to the number of residues in the data set.

According to a variable threshold, the prediction examples are classified as positives or negatives, and according to the target values, the predictions can be true or false. The predictions can be either true positives (TP), true negatives (TN), false positives (FP) or false negatives (FN).

The prediction accuracy is measured by constructing Receiver Operational Characteristics, ROC, curves [27]. For every value of the threshold, the true positive proportion, TP/(TP+FN), and the false positive proportion, FP/(FP+TN), is calculated. A ROC-curve is constructed by plotting the false positive proportion against the true positive proportion for all values of the threshold. It is therefore a non-parametric measure.

The sensitivity is equal to the true positive proportion, and the specificity, given by TN/(FP+TN), is equal to 1 – the false positive proportion. In this way, a ROC-curve is displaying the trade-off between the sensitivity and the specificity for all possible thresholds. A good method has a high true positive proportion when it has a low false positive proportion. A such model has a high sensitivity and a high specificity. The performance of the method is measured as the area under the curve, the *A*_*roc*_-value. For a random prediction, the true positive proportion is equal to the false positive proportion for every value of the threshold. Then *A*_*roc *_= 0.5. For a perfect method, *A*_*roc *_= 1.

### Bootstrapping

Bootstrapping is used to estimate the standard error of the *A*_*roc*_-value,  as a measure of the uncertainty of the *A*_*roc*_-value [28]. The relation between the standard error and the standard deviation, *s*, is that *se *= , where *r *is the number of repeats of the underlying experiment [29].

Bootstrapping is a method for generating pseudo-replica (bootstrap samples) of the predictions, denoted x*, which deviate a little from **x**. The bootstrap sample, x* = , is defined as a random sample of size *N*, drawn with replacement from **x**. Some of the prediction examples from **x **may appear zero times, some one time, some twice etc. Drawing a bootstrap sample can in other words be done by copying randomly chosen prediction examples, *x*_*n*_, from **x **into x*. In this way, some variation from **x **is introduced into x*.

Totally *B *bootstrap samples are drawn. Let x^*b ^denote the *b*'th bootstrap sample. The prediction accuracy of x^*b ^is calculated as .

The result of the bootstrap experiment is x^*1^, x^*2^,...,x^*B ^and hence . The standard error of the original *A*_*roc*_-value is given by



where  is the expected value of , given by [28]. Note the similarity to the way the standard deviation is calculated.  approaches the original *A*_*roc*_-value as *B *gets large.

### Paired t-tests

A paired t-test is performed in order to determine if one method is more accurate than another. The *H*_0_-hypothesis for this test is that two means are equal, *μ*_1 _= *μ*_2_. Instead of *μ*,  and hence *A*_*roc *_is used. The starting point is the performance measures of the two methods, *A*_*roc*,*M*1 _and *A*_*roc*,*M*2_, where *M1 *denotes method 1. By bootstrapping we have the vectors  and . Every bootstrap pair  are drawn identically for every *b*, making the two *A*_*roc*_-values paired.

The *H*_0_-hypothesis is therefore *A*_*roc*,*M*1 _= *A*_*roc*,*M*2 _and the alternative hypothesis *A*_*roc*,*M*1 _> *A*_*roc*,*M*2_. The test statistic *t *is given by



The paired difference of the *b*'th bootstrap samples, *D*^*b*^, is given by



The variable  is calculated as the expected value of *D*^*b*^, and  is calculated using (4) but replacing  with *D*^*b*^. The test statistic is following a t-distribution with *m *= *B *- 1 degrees of freedom, which approaches the normal distribution for *m *> 30, then *t *≈ *z*. The P-value for the test is then given by 1 - *F*(*z*), where *F*(*z*) is the cumulative normal distribution. See [29] for more information about the paired t-test.

### Permutation tests

When testing the *H*_0_-hypothesis that a method performs like a random model, a permutation experiment can be made. The alternative hypothesis is that the method is performing better than a random model. From the predictions of the method, **x**, the target values are permuted to result in a new prediction set, **x**^*perm,p*^. This is done for *p *= 1...*p*_*max*_. For every *p*, the prediction accuracy is calculated as . The P-value for the *H*_0_-hypothesis is calculated as the proportion of times for which  > *A*_*roc*_.

## Competing interests

The author(s) declare that they have no competing interests.

## Authors' contributions

JEPL collected the AntiJen and the HIV database, developed, tested and validated the prediction methods and drafted the manuscript. OL created the Pellequer database. MN implemented the programs for the prediction methods. All authors read and approved the final manuscript.
